# 

**DOI:** 10.1038/sj.bjc.6600374

**Published:** 2002-01-02


**WEEKLY PACLITAXEL IN OVARIAN CANCER IN THE NORTHERN IRELAND CANCER CENTRE – A SINGLE INSTITUTION EXPERIENCE.**

Kenny LM*, McDermott U, Atkinson RJ. N.Ireland Cancer Centre, Belfast, UK.

Aims: To retrospectively study the efficacy and toxicity of weekly paclitaxel in patients with a diagnosis of recurrent epithelial ovarian cancer who had previously received platinum based therapy.
Methods: A retrospective audit was performed of 40 patients with a diagnosis of recurrent ovarian cancer who were treated with weekly paclitaxel. The median follow-up was 12 months. The objectives of the audit were to determine progression-free survival, toxicity and cost. Patients received 80mg/m$^2$ of weekly paclitaxel and were treated to progression or development of grade 3/4 toxicity (NCI CTC version 2). The median number of cycles was 8 (range 1-34). All patients were assessed weekly. Response was evaluated by CT scan every 8-12 weeks and by regular measurement of serum Ca 125.
Results: The mean patient age was 58 (range 44-80yrs). Eighteen patients (45%) were optimally debulked at time of initial surgery. Six patients discontinued treatment due to toxicity before completing 5 cycles and were not evaluated for response. Of the remainder, 19 (48%) of patients had partial response/stable disease and the median progression-free survival was 8.5 months. The mean number of previous chemotherapy regimens was 2 (range 1-5). All patients had received previous platinum chemotherapy. 71% of patients had received previous paclitaxel treatment. There was some evidence of response in patients who had a paclitaxel (21 day regime) free interval under 6 months. The median time from previous paclitaxel (21 day regime) was 11 months, and from previous platinum was 7 months. At time of analysis 27 patients had died. All patients were assessed for toxicity (see below). There were no treatment-related mortalities. The mean cost of weekly paclitaxel per patient was £5730, as determined by the mean BSA.

| TOXICITY | Grade 1 | Grade 2 | Grade 3 | Grade 4 |
|---|---|---|---|---|
| NEUROLOGICAL | 4(10%) | 6(15%) | 1(2.5% | 0(0%) |
| ANAEMIA | 8(20%) | 11(27.5%) | 3(7.5%) | 0(0%) |
| NEUTROPENIA | 4(10%) | 8(20%) | 0(0%) | 0(0%) |
| THROMBOCYTOPENIA | 1(2.5%) | 0(0%) | 0(0%) | 0(0%) |
| MUSCULOSKELETAL | 3(7.5%) | 3(7.5%) | 1(2.5%) | 0(0%) |

Conclusions: Weekly paclitaxel is an active and well tolerated treatment for platinum pretreated patients with recurrent ovarian carcinoma, regardless of the paclitaxel-free interval. Only 12% of patients had grade 3 toxicity, and there was no grade 4/5 toxicity. The overall response rate of 48% is favourable compared to other regimens in this patient group.

**P35**

**A PHASE II STUDY OF SEQUENTIAL CARBOPLATIN, PACLITAXEL AND TOPOTECAN IN PATIENTS WITH PREVIOUSLY UNTREATED ADVANCED OVARIAN CANCER.**

*A.E.Guppy [1], A. Nelstrop [1], R. Agarwal [2], M.J. Seckl [2], and G.J.S.Rustin [1]
[1] Mount Vernon Hospital, Rickmansworth Rd, Northwood, Middlesex UK
[2] Charing Cross Hospital, Charing Cross Rd, London UK.

**Objective:** To evaluate the sequential use of carboplatin, paclitaxel and topotecan in patients with advanced, previously untreated ovarian cancer.
**Method:** Patients with advanced ovarian cancer and > 1 cm residual disease were treated with sequential Carboplatin (AUC5 days 1 and 22), Paclitaxel (175mg/m2 days 43 and 64) and Topotecan (1.5mg/m2 daily for 5 days from days 85, 106, 127 and 148). Disease response was assessed with CA125 and CT at cycles 2, 4, 6 and 8 and cycles 4 and 8 respectively.
**Results:** From 43 patients the median age was 61 years. Median follow-up was 17.3 months (range 0.76 – 42.3 months). Of a total of 310 cycles of chemotherapy, 19 cycles were delayed (15 patients) and dose modifications were necessary in 14 cycles (10 patients). 34 (79%) patients received all 8 cycles of treatment and 9 (21%) withdrew (4 due to progression and 2 due to toxicity). 19/28 (67.8%) evaluable patients responded according to RECIST and 30/36 (83.3%) patients to CA125. The best combined response was 76.7% (33/43 patients). The response rate to individual drugs based on > 50% fall in CA125 was as follows: Carboplatin, 76.9% (30/39 patients), Paclitaxel, 65.2% (15/23 patients), Topotecan, 38.4% (5/13 patients). Three patients responded after failure to respond to preceeding chemotherapy. The median survival was 21.81 months. Median time to progression was 10.58 months.
Conclusion: This study demonstrates that two courses of carboplatin followed by sequential paclitaxel and topotecan is an active and well tolerated first line regime in poor prognostic ovarian cancer patients. Overall response rates are equivalent to combination chemotherapy and individual response rates appear additive. This approach should help introduction of new agents.

**P36**

**NEOADJUVANT CHEMOTHERAPY AND SURGERY VERSUS STANDARD RADIOTHERAPY FOR LOCALLY ADVANCED CERVIX CANCER: A META-ANALYSIS USING INDIVIDUAL PATIENT DATA FROM RANDOMISED CONTROLLED TRIALS (RCTS).**

[1]LA Stewart*, [1]JF Tierney, for the Neoadjuvant Chemotherapy for Cervix Cancer Meta-analysis Collaboration (NACCMA Collaboration).
[1]MRC Clinical Trials Unit, 222 Euston Road, London NW1 2DA, UK.

The NACCMA Collaboration initiated a systematic review and meta-analysis to assess whether combined neoadjuvant chemotherapy and surgery is more effective than the standard radiotherapy approach to treating locally advanced cervix cancer. These preliminary results are based on updated individual patient data from 5 RCTs, conducted world-wide, that compared neoadjuvant chemotherapy plus surgery with radiotherapy alone. 872 patients (96% of known randomised patients) and 368 deaths were included.

| Outcome | HR (95%CI) | Absolute Effect at 5 Years (95% CI) | Effect P-value |
|---|---|---|---|
| Survival | 0.64 (0.52-0.79) | 15% (from 8 to 21%) | 0.00003 |
| Overall progression-free survival | 0.68 (0.56-0.82) | 13% (from 7 to 19%) | 0.00008 |
| Distant progression-free survival | 0.64 (0.52-0.78) | 15% (from 9 to 21%) | 0.00002 |
| Local progression-free survival | 0.67 (0.55-0.82) | 14% (from 7 to 19%) | 0.00008 |

The overall results showed a highly significant benefit of neoadjuvant chemotherapy combined with surgery compared to radiotherapy alone, with a 36% reduction in the risk of death. This is equivalent to an absolute improvement of 15% at 5 years, increasing survival from 45 to 60%. This survival improvement is comparable to that seen in the concomitant chemotherapy and radiotherapy trials. Similar results were obtained for the other outcomes, although there was some statistical heterogeneity (largely contributed by one of the trials). There was no good evidence that patient subgroups defined by age, stage, histology, grade or performance status, benefited more or less from neaodjuvant chemotherapy and surgery. These **preliminary results** suggest that neoadjuvant chemotherapy in addition to surgery may be of substantial benefit in patients with locally advanced cervix. An **updated analysis** will be presented.

© 2002 Cancer Research UK

ABSTRACT WITHDRAWN

**P37**
**AN EVALUATION OF PATIENTS WITH PERSISTENT GESTATIONAL TROPHOBLASTIC DISEASE (PGTD) WITH LUNG METASTASES ONLY ON CHEST X-RAY OR CT SCAN OF THE CHEST, IS THERE EXTRA BENEFIT FROM CT SCAN?**
SY Abdallah*, J Everard, CE Ingram, R Nakielny, RE Coleman and BW Hancock
Trophoblastic Disease Centre, YCR Academic Unit of Clinical Oncology, Weston Park Hospital, Sheffield, UK

Background: The world Health organisation (WHO) scoring of PGTD depends on several factors which include the number and site of metastatic lesions. CXR is used to assess lung metastases. The aim of this review is to evaluate whether the CT scan of the chest affected the WHO score and consequently the type of chemotherapy given to those patients.
Methods: Consecutive cases of PGTD with lung metastases detected by either CXR or CT scan of the chest were reviewed to assess whether the WHO score and hence treatment would have changed had CT scan findings been taken into account.
Results: Of 75 cases reviewed, in 21 chest CT scan showed lesions which were either not present or different from CXR findings; 5 patients would have had a different WHO score if the CT scan had been used in the scoring process. However none would have had a different chemotherapy regimen.
Conclusion: Although CT scan of the chest gives more information about lung involvement in PGTD, it has little effect on WHO score and consequently on the type of chemotherapy used in the treatment of those patients.

**P38**
**QUANTIFICATION OF ANGIOGENESIS IN PRIMARY AND METASTATIC EPITHELIAL OVARIAN CARCINOMAS**
*L.F. Wong Te Fong, G.K. Siddiqui, S.J. Gammell, M. Kini, [1]J.C. Crow, [2]E.S. Bamberger, W.M.N. Reid, A.B. MacLean, C.W. Perrett.
Departments of Obstetrics and Gynaecology and [1]Histopathology, Royal Free and University College Medical School, Royal Free Campus, Rowland Hill Street, LONDON NW3 2PF, U.K; [2]Department of Biology, University of Halifa, Oranim, Tivon 36006, Israel.

***Background:*** Angiogenesis, the formation of new blood vessels, is of crucial importance for tumour growth and development of metastases.
***Objectives:*** The purpose of this study was to quantify a primary regulator of angiogenesis, vascular endothelial growth factor-A (VEGF-A), in epithelial ovarian carcinomas (EOC) and in omental metastases, and to determine whether its expression had prognostic significance with survival.
***Study design:*** Formalin fixed, wax-paraffin embedded ovarian tissues of 28 primary tumours (Stages I-II, n=11; III-IV, n=17; grade 1, n=5; 2, n=13; 3, n=10) and 13 omental metastases were stained with a monoclonal antibody for VEGF-A, using immunohistochemistry. The quantification of VEGF-A was done according to the number of focally stained areas (each containing ≥ 10 cells) per $cm^2$ of the tissue section. Sections were defined positive if ≥ 10 focal areas/$cm^2$ were found in both the tumour area and the stroma. Statistics included non-parametric analysis (Mann Whitney U test) and survival analysis (Kaplan-Meier) with $p< 0.05$ considered significant.
***Results***. VEGF-A was expressed in 75% (21/28) of the primary tumours and in all 13 (100%) of the omental specimens, with a significant difference being found between early (46%) and late stage (89%) primary EOC. Serous primary tumours as well as grade 3 ovarian cancers of any type, showed higher VEGF-A expression, compared to other groups. The survival rate of patients with VEGF-A positive tumours was significantly worse than that of patients with VEGF-A negative tumours in EOC, during the first five years.
***Conclusion.*** These findings are consistent with the hypothesis that VEGF-A plays an important role in ovarian cancer related-angiogenesis and tumour progression and may have prognostic significance in terms of metastasis and survival.

**P39**
**CHARACTERISATION OF THE CELL CONTENT OF MALIGNANT ASCITES IN ADVANCED EPITHELIAL OVARIAN CANCER AND CLINICO-PATHOLOGICAL CORRELATIONS**
L Wall[1*], F Burke[2], J Smyth[1], F Balkwill[2]
[1] ICRF Medical Oncology Unit, Western General Hospital, Edinburgh. EH4 2XU
[2] ICRF Translational Oncology Unit, Barts and the London School of Medicine and Dentistry, Charterhouse Square, London.

**Aims**
The aim of this study was to characterise the cellular population of ascites in human epithelial ovarian cancer, and to correlate the cellular infiltrate with clinico-pathological patient details.
**Methods**
Ascites was collected from patients with advanced ovarian cancer at the time of therapeutic paracentesis or surgery. The cells from the ascites were used to make cytospins, which were stained with a panel of monoclonal antibodies to determine the nucleated ascitic cellular composition.
**Results**
We analysed 30 samples from 23 patients. Ascitic cellular composition fell into two main patterns, predominantly tumour cells (50% of samples) and predominantly small lymphocytes (40% of samples). The other patterns observed were predominantly fibroblasts (one sample) and predominantly granulocytes (two samples), despite the absence of clinical peritoneal infection. We found no relationship between the proportion of the various cell types and histological subtype, stage at diagnosis or prognosis.
Sequential sampling from a small number of patients suggested that response to chemotherapy was associated with a reduction in the proportion of tumour cells within the ascites. A greater proportion of mature macrophages was present in the ascites of patients who responded to chemotherapy than in non-responders (9.8% of cells versus 3.0%), although this was not statistically significant. Patients on chemotherapy had significantly more fibroblasts in the ascites (11.7% versus 4.0%), irrespective of subsequent treatment response.
**Summary**
There are two predominant cellular patterns within ovarian cancer ascites: a predominant lymphocyte infiltrate and a predominant tumour cell infiltrate. The inclusion of patients at different stages of their disease and treatment into our study did not allow us to determine whether the disease course of these patients differed.

Within individual patients there was some suggestion that ascitic composition reflected treatment status and outcome.

**P40**

**DETERMINING THE RESPONSE OF GYNAECOLOGICAL TUMOURS TO CHEMOTHERAPY USING AN MTS CYTOTOXICITY ASSAY IN COMBINATION WITH AN EXPLANT CULTURE TECHNIQUE**

*Sharon A. O'Toole, Brian L. Sheppard, Eamon P.J. McGuinness, Noreen C. Gleeson, Misaho Yoneda, John Bonnar.

Trinity College Department of Obstetrics and Gynaecology, University of Dublin, Trinity Centre for Health Sciences, St. James's Hospital, Dublin 8, Ireland.

The aim of this study was to use the (3-(4,5-dimethylthiazol-2-yl)-5-(3-carboxymethoxyphenyl)-2-(4-sulfophenyl)-2H-tetrazolium, inner salt) (MTS) assay to determine the response of malignant gynaecological tumours to various cytotoxic drugs. Malignant tumour samples of the ovary, cervix and endometrium were taken at surgery and cultured using the explant technique. Cells were reseeded and incubated with various concentrations of chemotherapy drugs. The MTS cytotoxicity assay was carried out to ascertain the response to the drugs and correlated retrospectively to the clinical outcome. Tumours of similar stage and grade displayed heterogeneity in their responses to the drugs. 88 of 90 tumours (97.8%) yielded chemosensitivity data. Of these, 45 were evaluable for *in vitro-in vivo* correlations. *In vitro* sensitivity was associated with clinical response in 26 of 30 patients, suggesting 87% prediction accuracy for sensitivity. *In vitro* resistance was associated with progressive disease or death in 14 of 15 patients, suggesting 93% prediction accuracy for resistance. The association between *in vitro* and *in vivo* results for all correlations, as measured by chi-square, was highly significant ($p<10^{-6}$). The sensitivity [(true positives)/(true positives + false negatives)] of the assay was 0.96. The specificity [(true negative)/(true negative + false positives)] was 0.78. A chemosensitivity / resistance profile of the tumour is recommended prior to deciding chemotherapy treatment.

**P41**

**PREDICTING PROGNOSIS IN UNRESECTABLE HEPATOCELLULAR CARCINOMA**

Ikram A. Burney*, Abdul Salam, Sarwar H. Orakzai, Raza H. Orakzai, Saleem. Sharieff.

Department of Medicine, Aga Khan University, Karachi, Pakistan.

The treatment outcomes of HCC remain dismal. Evolving experimental strategies and TACE offers hope for palliation. It may be prudent to select patients for these procedures. A retrospective analysis was undertaken to study the prognostic factors in relation to outcomes of unresectable HCC. Consecutive patients with histologically proven HCC and admitted to the Aga Khan University Hospital between January 1993 and December 2000 were the subjects of this study. A total of 348 cases were identified for analysis. The mean age was 57.4 ± 11.3 years. There were 251 males and 97 females. 43% had clinically overt jaundice at presentation. The Hepatitis viral profile was as under: Hep. B sAg +ve and Anti – HCV +ve = 4.9%; Hep. B sAg +ve only = 23%; Anti-HCV +ve only = 35.1%; Both negative = 14.9%; Not available = 22.1%. Laboratory investigations revealed: Hb = 11 ± 2.2 g/dl; TLC 11.3 ± 7.7 × $10^9$/L; platelet 210 ± 134 × $10^9$/L. bilirubin 4.8 ± 7.2 (0.1 – 51.5) mg/dl; SGPT 85.8 ± 11.83 (2 – 1577) IU/L; alkaline phosphatase 240.9 ± 241.5 (34 – 2750) IU/L; albumin 2.5 ± 0.7 g/dl; α - FP 15,345 ± 59,080 (0.7 – 712,600) ng/ml. α - FP was within normal limits for 24.5% of patients. The Radiological examination of the abdomen revealed: unifocal / multifocal lesions 49.4/50.6%; mean tumor size 7.7 ± 3.7 cm; extra-hepatic disease 19.8%; portal vein thrombosis 20.1%. One hundred and eleven (31.9%) patients received specific treatment. The median survival for the entire group of patients was 4 months, α - FP > 400 ng/ml, creatinine > 2 mg/dl, encephalopathy grade 2-4 and Okuda stage III were seen to independently predict worse survival on a multivariate analysis. A high α - FP was the most commonly encountered (47%) poor prognostic factor. Based on the four adverse prognostic factors a risk-based model was constructed. The median survival for 0 risk factors was 24 weeks, for 1 risk factor was 16 weeks and for 2-4 risk factors was 8 weeks ($p < 0.0001$). Based on this model, patients could be selected for the more aggressive palliative procedures such as TACE, and for the experimental studies.

**P42**

**EAST MEETS WEST: A COMPARISON OF OESOPHAGEAL CARCINOMA FROM HONG KONG AND SCOTLAND USING COMPARATIVE GENOMIC HYBRIDISATION**

SC Stocks [1] M Sales [2]VGoldie*[3] N Pratt [2], F Carey[1] 1, N Kernohan[1] 1, R Stuart[4], S Chung[5], A Chan[5] , D Johnston[1] , AM Thompson [3].

Departments of Molecular and Cellular Pathology[1], Human Genetics[2] and Surgery and Molecular Oncology[3], University of Dundee, Dundee, DD1 9SY; [4]Department of Surgery, University of Glasgow and [5]Chinese University, Prince of Wales Hospital, Hong Kong.

**Aim**: Little is known of the precise sequence of genetic events that define malignancy or underlie the clinical course of squamous oesophageal carcinoma. In this study we sought to compare the range of genetic aberrations in two geographically distinct populations.

**Methods**: Comparative Genomic Hybridisation (CGH) was performed on resected specimens from 41 patients: 18 cancers resected in Hong Kong and 23 cancers resected in Scotland. The histology was confirmed as squamous carcinoma in all patients. For comparison and as control cancer samples the oesophageal cancer cell lines KYSE-30 and OE21 were used.

**Results**: Genetic aberrations were detected in 14/18 Hong Kong cancers and 18/23 Scottish cancers. Deletion of 3p, 4p and 8p were identified in 3 or more cancers from both geographical sites, but in addition, 5q and 18q deletions were identified in 3 or more of the Scottish cancers. Similarly, while amplification of 1q, 3q, 5p, 8q, 9q and 17q were found in the Hong Kong cohort, additional 7p and 7q, 14q and 18p amplifications were also identified in 3 or more Scottish cancers. However, these additional CGH amplifications/deletions were also, rarely, identified in the Hong Kong population. For some changes the CGH data are consistent with an underlying molecular pathology such as activation of myc (8q24), erb B2 amplification (17q12), and inactivation of tumour supressors APC (5q) and DCC (18q). In other instances, such as amplification of 1q and deletion of 8p, strong candidate genes have yet to be identified.

**Conclusion:** In keeping with the known common aetiologies, these data obtained using CGH suggest that squamous carcinoma of the oesophagus has a similar spectrum of amplifications and deletions in the East and West

**P43**

**IS POOR SURVIVAL FROM OESOPHAGEAL AND GASTRIC CANCER SURGERY DUE IN TO INADEQUATE STAGING?**

AM Thompson*, Department of Surgery and Molecular Oncology, University of Dundee, DD1 9SY for the Scottish Audit of Gastric and Oesophageal Cancer

**Aim**: To assess the staging of patients undergoing gastric or oesophageal resection.

**Methods**: The population-based Scottish Audit of Gastric and Oesophageal Cancer (SAGOC) collected data prospectively from every patient diagnosed with oesophageal or gastric cancer in Scotland over a 2 year period (to September 1999). Follow up was continued for a minimum of 1 year after diagnosis. Data was analysed by the Scottish Cancer Intelligence Unit of the National Health Service.

**Results:** 1302/3292 (39.5%) patients identified over the 2 year period underwent surgery; 1006 had pathology data on a resected specimen. Resection margins were involved in 114/371 (31%) oesophageal resections, 44/156 (29%) junctional resections, and 95/479 (20%) gastric cancer resections, the majority of whom had pT3 or pT4 cancers. The radial (circumferential) margin was involved in 68% of patients with resection margin involvement. For oesophageal and junctional cancers, this was not related to the surgical approach used. Three quarters (180/240) of patients

© 2002 Cancer Research UK

with resection margin involvement also had nodal metastases (93% of those with multiple margin involvement). Given that the one year survival for the patients who underwent operation was 54%, these data suggest that operative intervention was inappropriate in upto a third of patients who underwent surgery.
Although 93% patients underwent CT of chest and abdomen prior to oesophagectomy with curative intent, only 30 (8%) underwent endoscopic ultrasound (EUS). For junctional cancers, 90% had an abdominal CT and 85% a CT chest but laparoscopy was used in only 44% and EUS in 6%. For gastric cancer, 71% underwent abdominal CT, 33% laparoscopy and 4% EUS. Preoperative staging was therefore incomplete in a substantial proportion of patients.
**Conclusions:** To avoid inappropriate (non-curative) surgery, improvements in the provision (for EUS) and utilisation (for laparoscopy) of staging tests and their correct interpretation (of, for example, CT scans) will be required. These data suggest that major changes in clinical practice are needed to improve the selection of patients for curative oeosphageal and gastric surgery.

**P44**
**OESOPHAGEAL CANCER CELL LINES APOPTOSE FOLLOWING TRANSFECTION WITH WILD TYPE P53.**
Oglesby SD[1]*, Hupp TR[2], Thompson AM[1]. Departments of Surgery & Molecular Oncology[1], and Molecular & Cellular Pathology[2], University of Dundee, Dundee. DD1 9SY.

Introduction. Most patients with oesophageal cancer do not undergo surgery and many are treated with radiotherapy. We have previously reported that the oesophageal cancer cell lines OE19, OE21, and OE33 have p53 mutations, and that these mutations lead to resistance to this treatment modality (Oglesby et al, BCRM 2001). We sought to assess the effect of restoring functional p53 in these cell lines by means of liposome mediated transfection of plasmid containing wild type p53.
Methods. SV53 is a plasmid which constitutively expresses wild type p53 via an SV40 promoter. OE19, OE21, OE33 were each grown in 75cm$^2$ tissue culture flasks until 70% confluent at which point they were transfected with SV53. TFX-50 (Promega) a liposomal transfection reagent was used to transfect 15μg plasmid in each flask. Cells were collected by trypsinisation at 6 hourly intervals from 0 to 24 hours. OE19 and OE21 have p53 mutations which lead to production of a truncated protein. Expression of full length protein in these cell lines can therefore be assessed by western blot analysis. Assessment of apoptosis was performed by flow cytometry using propidium iodide.
Results. In the OE19 and OE21 cell lines, expression of full length p53 was detected at 6 hours and was maximal at 18 hours. All cell lines showed increasing induction of p21 over the 24 hour time course. Apoptosis was identified at 6 hours, and was maximal at 24 hours.
Discussion. Our study shows that it in cell lines which are resistant to ionising radiation, transfection with wild type p53 is very effective at inducing programmed cell death. Given that the p53 mutation rate in oesophageal cancer may be as high as 84% (Robert et al, Carcinogenesis 2000), and that most oesophageal cancers exhibit limited sensitivity to conventional treatment, restoration of functional p53 by transfection may provide a useful therapeutic strategy in this disease.

**P45**
**P 53 MUTATION RATE IN OESOPHAGEAL CARCINOMA IS HIGHER THAN GENERALLY ACCEPTED**
Oglesby SD[1]*, Warbrick E[1], Johnston DA[2], Dillon JF[2], Munro AJ[1], Hupp TR[2], Thompson AM[1]. Departments of Surgery & Molecular Oncology[1], and Molecular and Cellular Pathology[2], University of Dundee, Dundee. DD1 9SY.

Introduction. p53 is a key regulator of cellular response to radiation and cytotoxic drugs. The currently accepted p53 mutation rate in oesophageal carcinoma is in the region of 50% (Beroud et al, Nuc Acid Res 1998). Until recently, sequencing has been the gold standard for identifying inactivating mutations. However, sequencing can perform poorly when used to analyse material with a mixture of cell types such as found in tumour biopsies. The functional yeast assay (FYA) is a relatively new method of evaluating p53 mutations and outperforms sequencing when used on biopsy material. Duddy et al (J Mol Diagn 2000) found that sequencing p53 exons 5-8 identified only 54% of mutations detected by FYA in breast cancer. Robert et al (Carcionogenesis 2000) analysed 50 squamous and 6 adenocarcinomas of the oesophagus using FYA and report a mutation frequency of 84%. However, in most western societies, the frequency of adenocarcinoma now exceeds squamous. We sought to identify the frequency of p53 mutations in carcinoma of the oesophagus in a typical western population.
Methods. Tumour biopsies were obtained from 30 consenting patients with oesophageal carcinoma. The biopsies were assessed for p53 mutations using the FYA. Confirmation of p53 mutation was sought by sequencing plasmid recovered from single yeast colonies. The histological tumour type was recorded.
Results. 22 of 30 tumours were adenocarcinomas, all were identified as containing mutant p53 by FYA. The remaining 8 tumours were squamous and 4 of these (50%) were mutant. The overall p53 mutation rate was 87%, all mutations were confirmed by plasmid sequencing. All mutations were within the core DNA binding domain of p53.
Discussion. Our study confirms a high p53 mutation frequency in oesophageal carcinoma and also shows that this applies to adenocarcinomas. Previous studies attempting to correlate p53 status with treatment responsiveness have been inconclusive, which may in part be due to underreporting of p53 mutations.

**P46**
**ORAL CAPECITABINE IN METASTATIC COLORECTAL CANCER (CRC)**
B.Basu*, J.Abraham, V.R.Bulusu, K.Grant, M.Corcoran, J.Shaw, R.Harris, P.Corrie, C.B.Wilson, Oncology centre, Addenbrooke's hospital, Cambridge, UK CB2 2QQ.

**Introduction:** Capecitabine is one of oral 5FU prodrugs, which is activated preferentially in the tumour. We present the 'real life' data in patients (pts) with metastatic crc.
**Aim:** To assess the overall response rates and toxicity profile of oral capecitabine in pts with metastatic crc.
**Material and Methods:** 18 pts (9 males, 9 females) with metastatic crc were treated with oral capecitabine at 2.5gm/m$^2$ /day for 14 days, cycle repeated every 3 weeks. Mean age 64 years, WHO performance status 0-2. All had normal haematology and renal function. No dose corrections were made for hepatic dysfunction. 18 pts are evaluable for toxicity and 17 for response. Overall response rate (ORR), complete response (CR), partial response (PR) and stable disease (SD) were assessed using WHO criteria and toxicity as per the common toxicity criteria. Survival was calculated from the date of first cycle.
**Results:** A total of 98 cycles were administered with median of 5. 1 CR, 4 PRs ( ORR=29%) and SD in 9 (53%) were noted. Only one pt progressed on treatment. Grade 3 toxicity was noted in 44% of pts, 27% palmar-plantar erythema (PPE), 17% fatigue, 5% mucositis and diarrhoea. 1 pt required hospital admission for management of toxicity. 33% had dose reductions to a mean of 84% of the total projected dose. No treatment related deaths were recorded.
**Conclusions:** Oral capecitabine is well tolerated in pts with metastatic crc with an ORR of 29%. The major dose limiting toxicities were PPE and fatigue. No differences in the toxicity pattern between the sexes were observed. PPE responded to dose reductions/schedule alterations/pyridoxine.

**P47**
**EXTERNAL BEAM RADIOTHERAPY (EBRT) IN LOCALLY ADVANCED PANCREATIC CANCER (LAPC): IPSWICH EXPERIENCE**
V.R.Bulusu*, E.McEwan, L.Smith, A.J.Poynter, J.Crosbie, O.Allen, T.J.Podd, Suffolk Oncology Centre, Ipswich Hospital, Heath Rd, Ipswich, UK IP4 5PD.

**Introduction:** The role of radiation in LAPC is not clearly defined. Gemcitabine has been the standard chemotherapy for pancreatic cancer. We

© 2002 Cancer Research UK

present our experience with EBRT in LAPC treated at Ipswich hospital from 1999 to 2001.

**Aim**: To assess the overall survival and toxicity in patients who have received EBRT for LAPC.

**Material and Methods**: 21 patients (pts) who have received EBRT for LAPC were included in this study. Pancreatic cancer was diagnosed either by histology and/or CT criteria in 13 males and 8 females with a mean age of 63 yrs |(range 51-75years). All had WHO performance status 0-2. Prophylactic ranitidine or lansaprazole was prescribed from the start of EBRT. Majority of the pts received chemotherapy 5FU or gemcitabine either adjuvant or on progression.

Radiation Treatment Protocol: Pts were treated with either a parallel opposed fields (n=6) or 3 field CT plan (n=15) using 6/16 MV photons. Radiation dose was specified to the ICRU 50 recommended reference point. CT imaging was performed using either a Siemens Somatom CT scanner (n=18, slice width=10mm) or a GE Light Speed CT scanner (n=3, slice width=5mm). Fifteen pts were planned using the Nucletron PLATO treatment planning system.

**Results**: All 21 pts were assessable for both toxicity and survival. Only 1/21 failed to complete EBRT due to grade 3 gastrointestinal toxicity. Mean and median EBRT doses 44.5 and 50Gy (95% CI 40.5-48.5). Toxicity data: Nausea 100%, emesis requiring treatment 45%, diarrhoea 20% indigestion 10% and 5% weight loss. Kaplan-Meier survival estimates for 6, 9, 12 and 24 months are 0.90, 0.75, 0.55 and 0.22 respectively. 6/21pts are alive at 5,11.5,11.5,14.5,22 and 27 months from the date of diagnosis. There were no gut perforations or treatment related deaths. No Grade 4 acute toxicity was recorded.

**Conclusions**: EBRT for LAPC is well tolerated with acceptable toxicity. EBRT may have a role in the management of pts with LAPC. Recent advances in conformal/intensity modulated radiotherapy may help to achieve a better therapeutic ratio and may allow further dose escalation. Future trials in pancreatic cancer should critically evaluate the role of radiotherapy in improving local control and possibly overall survival.

### P48
### BIOPSY OF A BIOPSY: VALIDATION OF IMMUNOPROFILING IN GASTRIC CANCER BIOPSY TISSUE MICROARRAYS.

Christian Gulmann*, David Butler, Elaine Kay, Antoinette Grace, Mary Leader.
Department of Pathology, Beaumont Hospital and Royal College of Surgeons, Dublin, Ireland.

Background and aim: Tissue microarrays offer an efficient way of examining a large number of tumour cases on a single glass slide. A major concern, however, is tumour heterogeneity. Also, the use of tissue microarrays in biopsy material is unexplored. The purpose of the present study was to assess the possibility and validity of arraying three 0.6-mm cores per case in endoscopic gastric cancer biopsies for immunophenotyping.

Materials and methods: Thirty-eight cases were studied with immunohistochemical staining for p53, CD44v6 and vascular endothelial growth factor. All play a role in the progression of gastric cancer and were chosen to cover a spectrum of complexity of staining interpretation as well as staining different cellular substructures. p53 was interpreted as positive or negative whereas the two other stains were graded for intensity and extent with chosen cut-off points for overall positivity/negativity. With triplet cores 'majority decisions' (i.e. two cores versus one) were carried out in the case of a discrepancy between cores from the same case. Full tissue sections were compared with triplet core-tissue microarrays.

Results: Thirty-six cases contained three cores with tumour, one case contained two cores with tumour and one case contained only a single core with viable tumour and was excluded. Three further cores had been lost from 3 separate cases on the sections for immunohistochemistry. Concordance rates and kappa-values were 97% and 0.94 for p53, 92% and 0.77 for CD44v6 and 97% and 0.77 for vascular endothelial growth factor. p53 immunohistochemical staining yielded the strongest result with only 1/37 mismatches, whereas CD44v6 showed 3/37 mismatches.

Conclusion: Tissue microarray with three cores per case is a feasible and valid way of studying biopsy material. The small depth of tissue in cores from biopsies necessitates all cores being arrayed flush with the face of the recipient wax block for maximising number of sections available. It is therefore impractical to array more than 120-150 cores per block.

### P49
### A PROSPECTIVE CASE CONTROL ANALYSIS OF CYCLOOXYGENASE-2 EXPRESSION IN BARRETT'S OESOPHAGUS PATIENTS

J.Jolly[1*], L.J.Hardie[1], M.F. Dixon[2], C.P.Wild[1].
[1]Molecular Epidemiology Unit, School of Medicine, University of Leeds and [2]Department of Pathology, Leeds General Infirmary, Leeds LS2 9JT.

One of the greatest risk factors for the development of oesophageal adenocarcinoma (AO) is the presence of Barrett's oesophagus (BO). This condition is characterised by the replacement of the normal squamous epithelium of the oesophagus with a specialised, columnar type. Molecular alterations which occur during the development of BO, may serve as biomarkers to assess the risk of AO development in this patient group (*Bani-Hani et al., 2000; JNCI 92; 1316-1321*). The prostanoid synthesizing enzyme, cyclooxygenase-2 (COX-2) is reported to be upregulated in BO and AO, and epidemiological studies link this enzyme to AO development through the observed protective effect of non-steroidal anti-inflammatory drug use.

The aim of this study was to examine the potential predictive utility of COX-2 expression as a molecular marker of AO development in BO patients.

A total of 307 BO patients were enrolled into an endoscopic surveillance cohort at Leeds General Infirmary between 1984-1995. For each incident case of adenocarcinoma, four control patients were matched for duration of follow up, age, sex, and length of columnar epithelium at recruitment. Following ethical approval, biopsies obtained from cases at recruitment and at diagnosis of AO, together with matched biopsies from control patients were stained for COX-2 expression using conventional immunohistochemistry techniques. Slides were assessed in a blinded fashion by a pathologist using a semi-quantitative scoring system.

COX-2 was expressed in all of the AO tumours (N=15) arising within the cohort. However, biopsies taken at recruitment from the same incident AO cases were no more likely to be COX-2 positive (40%) than those of matched control patients (55%). However, the number of biopsies which expressed COX-2 increased (55% - 80%) with duration of follow-up in control BO patients. In addition, a greater proportion of follow-up biopsies stained for COX-2 in both surface and glandular compartments compared with recruitment biopsies in these patients.

In this study, no difference was observed in COX-2 expression between cases and controls at recruitment, suggesting that COX-2 is not informative at identifying BO patients at increased risk of progression to AO. However, that COX-2 expression was observed to increase and change in localisation with time, is suggestive of an association with disease development and warrants further investigation.

### P50
### INCREASE IN GENE AND PROTEIN EXPRESSION OF GASTRIN, CCK2R, MMP-2 AND TIMP1 IN BARRETT'S COMPARED TO PAIRED NORMAL SAMPLES

JC Harris[*1], RA Dean[1], PA Clarke[1], A Awan[1], J Jankowski[2], SA Watson[1]
[1]Academic Unit of Cancer Studies, QMC, University Hospital, Nottingham NG7 2UH; [2]Department of Medicine, University of Birmingham, Edgbaston, B15 2TH

**Background and Aims:** Barrett's Oesophagus is a pre-malignant condition of the lower oesophagus caused by prolonged gastro-oesophageal acid reflux and characterised by a metaplastic change from normal squamous to columnar intestinal-type epithelium. The aim of this on-going study was to assess levels of gastrin, cholecystokinin 2 receptor (CCK2R), and a variety of matrix metalloproteinases (MMPs) and tissue inhibitors of metalloproteinases (TIMPs) in normal and Barrett's biopsy samples and two established oesophageal cell lines.

**Methods:** Real-time PCR was used to investigate the gene expression of gastrin, CCK2R, MMP-2, -9, MT1-MMP, TIMP1 and 2, in the normal and Barrett's biopsy samples (n=9) as well as the cell lines OE19 (oesophageal adenocarcinoma, pathological stage III) and OE33 (oesophageal Barrett's metaplasia, pathological stage II). Immunohistochemistry was used to investigate progastrin and CCK2R at the protein level in all samples.

**Results:** The grade III adenocarcinoma cell line OE19, was shown to express relatively higher levels of gastrin, MMP-2 (P=0.05) and TIMP1 (P=0.0413) at the gene level compared to the Barrett's metaplasia cell line, OE33. These

 © 2002 Cancer Research UK

results correlated with the biopsy samples where higher levels of gastrin (P=0.0423), total CCK2R (P=0.0406), MMP-2 (P=0.031) and TIMP1 (P=0.041) gene expression was observed in Barrett's compared to normal epithelium. With regard to CCK2R, immunohistochemistry illustrated a stepwise increase in levels of the intron 4 splice variant (CCK2Ri4sv) isoform of the receptor, as normal tissue progressed to Barrett's (P=0.045).
**Conclusion:** The mRNA levels of gastrin, MMP-2 and TIMP1 have been shown to be higher in the adenocarcinoma cell line OE19, compared with the Barrett's metaplasia cell line OE33. Similar results were shown in the Barrett's metaplasia biopsies compared to their paired normal samples, including an increase in CCK2R gene expression. An increase in CCK2Ri4sv at the protein level has also been shown. It is hence concluded that progression to Barrett's Oesophagus could be associated with an increase in MMP, gastrin and CCK2Ri4sv expression.

**P51**
**GENE EXPRESSION AND CELL CYCLE ANALYSIS OF COLORECTAL CANCER CELL LINES EXPOSED TO 5FU AND 5FDURD**
Sandra Easdale[*1], Paul. Clarke[1], Jenny Titley[1], Richard Wooster[2] and Paul Workman[1]
[1]Cancer Research UK, Centre for Cancer Therapeutics, Institute of Cancer Research, 15 Cotswold Road, Sutton
[2]Institute of Cancer Research, 15 Cotswold Road, Sutton and the Sanger Centre, Cambridge

Although, 5FU is the mainstay treatment for colorectal cancer, its precise molecular mechanism of action is undefined. It is already known that 5FU treatment results in inhibition of thymidylate synthase and misincorporation of its metabolites into both DNA and RNA. Microarray technology allows for the measurement of global gene expression and is poised to revolutionise the discovery and use of new anticancer agents. This project involved the analysis of gene expression patterns in three human colorectal cancer cell lines exposed to 5FU and one of its important metabolites 5FdUrd. 5FU was chosen for this analysis due to its complex mechanisms of action that were expected to challenge the microarray technology and there has also been a long standing interest in the mechanism of action of 5FU and thymidylate synthase inhibitors within our Institution. The three cell lines were exposed to five times their $IC_{50}$ concentrations of 5FU or 5FdUrd and cell cycle distribution analysed over 2-72 hours. Messenger RNA was also extracted from cells exposed to the same drug concentrations and time course. The mRNA was labelled with fluorescent dyes (Cy3 and Cy5) using reverse transcription and hybridised to 5808 cDNA clones arrayed on glass slides. The array slides were prepared in-house and encompass known genes and ESTs known or suspected to be associated with cancer, for example cell adhesion genes, apoptosis genes and cell cycle genes. Cell cycle analysis showed that each cell line was arrested in the G1 phase of the cell cycle and a depletion of cells in S phase was also found. Each cell line was affected to a varying degree. For example, HCT116 cells demonstrated an accumulation of cells in late G1/early S phase at 4 hours post treatment, whereas both HT29 and SW480 cells did not exhibit this effect until between 16 and 24 hours. Both 5FU and 5FdUrd treatments cause similar effects upon the cell cycle in these three cell lines. Thymidine, but not uridine protected HT29 cells from the effects of 5FU and these observations indicate that the 5FU is acting via thymidylate synthase inhibition within these cell lines. Microarray analysis of 5FU and 5FdUrd treated cells demonstrated that each cell line had its own distinctive pattern of gene expression. A considerable number of genes exhibited altered expression, either increased or decreased relative to control levels. In excess of 500 genes were altered over the entire course of the experiment within each cell line. No evidence of alteration of genes involved in pyrimidine metabolism or DNA/RNA metabolism was found. From the array analysis both 5FU and 5FdUrd treatment appear to affect similar types of genes, for example those involved in the regulation of the cell cycle. No evidence for p53 induced gene expression could be found in wild-type p53 HCT116 cells contrary to published data at higher 5FU concentration. The analysis of our data is still ongoing; however preliminary results have indicated that several genes involved in the regulation of the cell cycle (for example RB, CIP2/KAP1 and CDK8) have altered expression.

**P52**
**PRELIMINARY RESULTS OF A PHASE II PILOT STUDY OF 13-CIS-RETINOIC ACID WITH GEMCITABINE IN PATIENTS WITH UNRESECTABLE PANCREATIC OR CHOLANGIOCARCINOMA.**
Michael A. *[1] Maraveyas A[2], Lofts F[1] ; 1-St. George's Hospital London SW17 0QT, 2-The Princess Royal Hospital Hull HU8 9HE

Patients with locally advanced or metastatic pancreatic cancer have a poor prognosis and suffer severe disease related symptoms. The median survival without any treatment is approximately 3 months. The one treatment that has proved beneficial and well tolerated is chemotherapy. Comparison between Gemcitabine and weekly 5FU gave a 5-week improvement in median survival. Gemcitabine also increases clinical benefit response as defined by improvement in pain, weight loss and performance status. Unresectable cholangiocarcinoma is a chemoresistant cancer with extremely poor prognosis. Patients with this condition should be considered candidates for inclusion in clinical trials.
The retinoids are derivatives of vitamin A that are known to affect tumour cells, can inhibit growth as well as induce differentiation and apoptosis. Pre-treatment of pancreatic adenocarcinoma cells with 9-cisRA followed by Gemcitabine resulted in a strong increase in apoptosis (Pettersson F, 2001: Pancreas, Vol.23, p.273). 13-cis retinoic acid has been studied in many solid tumours with some promising results in glioma and renal cell carcinoma (Aisner J. 2000, ASCO Abstracts No1416, Jaeckle 2000, ASCO Abstracts No 629).
We are conducting a study of Gemcitabine in combination with 13-cis retinoic acid in locally advanced or metastatic carcinoma of pancreas or cholangiocarcinoma. Gemcitabine is given on day 8,15 and 22 at a dose of $1000mg/m^2$ and 13-cis RA is given orally, day 1 to 14 at dose of 1mg/kg. To date eleven patients have been recruited (2F/9M), median age 63 (range 45-78) and median Karnofsky PS 93% (90-100%). Out of 20 cycles given only one case of grade 3 toxicity has been reported with thrombocytopaenia . The recruitment of this well tolerated regimen continues.

**P53**
**COLORECTAL CANCER CELL DEATH IN PATIENTS RECEIVING NEOADJUVANT IMMUNOTHERAPY – PRELIMINARY RESULTS**
AA Indar[1], M Dube[1], RA Robins[2], LG Durrant[3], J Carmichael[3], JH Scholefield[1]
Section of Gastrointestinal Surgery[1] and Department of Immunology[2]; University Hospital Nottingham, NG7 2YH; Academic Department of Clinical Oncology[3], Nottingham City Hospital, Nottingham, NG5 1PB.

**Background:** 105AD7 is an anti-idiotypic antibody that mimics CD55 found on 80% of colorectal cancer cells, and has shown effective antigen-specific immune responses in immunised colorectal cancer patients. We assessed apoptosis in this group of patients in a randomised control trial.
**Methods:** Patients with colorectal cancer were randomised to treatment with neo-adjuvant 105AD7/Alum, or 105AD7/BCG or control, with the treatment arms immunized 2-3 weeks prior to surgery then at regular intervals thereafter. Resected tumour specimens underwent 3-colour flow cytometric analysis with Apo2.7 and Anti-Caspase-3 antibodies to detect apoptosis.
**Results:** 12 patients receiving 105AD7/Alum, 14 who received 105AD7/BCG, and 16 control patients had their adenocarcinomas analysed (Dukes A-6, B-15, C1-16, C2-4, D-2). There were increased levels of Apo2.7 in patients receiving 105AD7/Alum compared to controls, but with a wide range of data. Proliferation was also reduced in the treated group.

| Medians | CONTROL | 105AD7/Alum | *p* value | 105AD7/BCG | *p* value |
|---|---|---|---|---|---|
| Tumour Apo2.7 | 13.5 | 20.6 | .66 | 11.8 | .51 |
| Tumour Anti-Caspase-3 | 2.56 | 2.79 | .98 | 1.22 | .98 |
| Tumour Ki-67 | 2.76 | 2.12 | .87 | 2.3 | .88 |
| Lymphocyte Apo2.7 | .43 | .36 | .39 | .51 | .48 |
| Lymphocyte Anti-Casp-3 | .03 | .02 | .31 | .02 | .34 |
| Lymphocyte Ki-67 | .13 | .16 | .35 | .16 | .31 |

5/6 patients receiving 105AD7/Alum, 2 receiving 105AD7/BCG and no control patients showed specific T cell proliferation responses, however only 2 of these patients responded to the initial pre-operative dose with both having high rates of tumour apoptosis. The other patients responded after their fourth booster.

**Conclusion:** Increased levels of apoptosis were seen in colorectal cancer patients receiving 105AD7/Alum compared to controls, however this sample is too small at present to draw any firm clonclusion, but recruitment is presently ongoing. This reflects the difficulty in measuring apoptosis in clinical practice where variable factors result in high background values. However 105AD7/Alum does induce good cellular immune responses that may result in improved clinical response in colorectal cancer patients.

**P54**

**AN EXTENDED PHASE II FEASIBILITY STUDY OF GEMCITABINE & CISPLATIN IN ADVANCED OESOPHAGEAL CANCER – INTERIM ANALYSIS**

J. Millar[1*], S. Clive[2], D. Dunlop[3], J.Godden[3], A. Price[2], D. Cameron[2], H. Philips[2], D. McGrath[1], A. Morrison[1], M. Eatock[1]. [1]Northern Ireland Cancer Clinical Trials Unit, Belfast City Hospital. [2]Edinburgh Cancer Centre, Western General Hospital, Edinburgh. [3]Glasgow Royal Infirmary, Glasgow.

<u>Introduction:</u> Gemcitabine (Gem) is active against a broad range of tumour types. Preclinical models suggest synergy between Gem and Cisplatin (Cis), and in phase II trials this combination has activity in NSCLC, head & neck cancer and bladder cancer. This study aims to assess its activity in oesophageal cancer.

<u>Methods:</u> Patients with histologically proven, bidimensionally measurable, locally advanced or metastatic oesophageal carcinoma (squamous or adenocarcinoma), aged >18yrs, with a performance status 0-2 and life expectancy > 3 months, who were able to give informed consent were eligible. A total of 48 patients are required to differentiate between response rates of > 60% and < 40% with a power of 80% at the 5% significance level.

<u>Treatment:</u> Treatment with Gemcitabine 1250 mg/m$^2$ on days 1 and 8, and Cisplatin 75 mg/m$^2$ on day 1 of a 21 day cycle was given. Gemcitabine dose was reduced by 50 % if, on the day of treatment, ANC 0.5 – 0.9 x $10^9$/L or plts 50 – 99 x $10^9$/L. Treatment was withheld if the ANC was < 0.5 and plts < 50. The Cisplatin dose was split over 2 days if calculated creatinine clearance was 40 – 60 ml/min, and omitted if < 40 mls/min. Treatment was stopped in the event of grade 3/4 peripheral neuropathy.

<u>Results:</u> Due to concerns over toxicity, an interim analysis was performed after 19 pts (M:F = 16:3. Median age = 60yrs (range 38 – 79 yrs)) were entered. One patient had received 6 cycles of Cis/5FU as adjuvant treatment 12 months earlier. Median number of cycles completed = 4 (range 1 – 6), with 11pts completing at least 4 cycles and 6 pts completing 6 cycles. Haematological toxicity in cycle 1 was as follows: Neutropenia – grade 4 in 2 pts; grade 3 in 6 pts; Thrombocytopenia – grade 3 in 2 pts; Anaemia – grade 4 in 1 pt (not treatment related). In all cycles, 13 pts had at least grade 3 haematological toxicity, but there were no episodes of neutropenic sepsis or complicated thrombocytopenia. The most common non-haematological toxicities were nausea & vomiting (grade 3/4 in 8 pts despite 5HT3 antagonist and steroid premedication) and lethargy (grade 3 in 5pts). 4 pts discontinued therapy due to toxicity. Partial responses were observed in 7 pts, with stable disease in a further 6.

<u>Conclusion:</u> This level of toxicity and dropout rate was felt to be unacceptable for a palliative treatment at the current doses. In evaluable pts a response rate of 41% was achieved which is comparable to Cis/5FU. In view of this, the trial will continue to its recruitment target with a reduced Gem dose and an altered dose modification schedule.

**P55**

**A WEST OF SCOTLAND AUDIT OF SHORT-COURSE PRE-OPERATIVE RADIOTHERAPY FOR OPERABLE RECTAL CANCER**

V Hughes* and AC McDonald, Beatson Oncology Centre, Western Infirmary Glasgow G11 6NT, UK.

**Introduction** Short-course (25Gy in 5 fractions, over 1 week) pre-operative radiotherapy (pre-op RT) reduces local failure and may improve survival in patients (pts) with operable rectal cancer, however it is not without toxicity. This audit was performed to determine the use of short-course RT in the Beatson Oncology Centre and to identify whether some pts may be better served by alternative approaches.

**Methods** A retrospective case sheet audit was undertaken of pts receiving short course pre-op RT for rectal cancer between June 1999 and December 2000. Patients were identified from the computerised RT booking system. Information from clinical notes and pathology reports was reviewed.

**Results** Forty-seven pts received short-course pre-op RT during the period studied, with data available on 44pts. Five pts received treatment within the MRC CR07 study (comparing short course pre-op RT with immediate resection); 2 further pts entering CR07 drew immediate surgery during the period audited. Baseline staging comprised clinical tumour assessment, abdomino-pelvic CT (38pts), CXR & liver ultrasound (4pts) and MRI (1pt). Eleven patients underwent trans-rectal ultrasound (uT2=2, uT3= 7, uT4=2). Clinical details were poorly documented, with rectal examination findings and tumour mobility detailed in only 27 and 20 pts respectively. Of these 20 pts, 8 were said to have fixed tumours at the time of referral. Treatment was delivered by 9 oncologists, however of these only 3 each treated more than 2 pts. Eight clinicians used a planned volume, however 1 used opposed AP-PA fields. All 44 pts completed treatment and underwent tumour resection, potentially curative in 41 cases. Median time to resection was 4 days, (range 1-13) after completion of RT. Pathologic staging identified Duke's A 8pts (18%), B 17pts (39%), C 16pts (36%) and D 3pts. Eleven pts (25%) had involvement (≤1mm clearance) of the circumferential resection margin (CRM). In 17pts (38%) there was no documentation of histopathologic findings in the case-sheet. There were no post-op (<30 days) deaths, but treatment toxicity and post-op complications were poorly recorded.

**Conclusion** Of 9 oncologists using short-course pre-op RT, the majority treated only one or two cases. A number of (Duke's A) patients at low risk of local failure received pre-operative treatment. The 11 pts with locally advanced disease and CRM involvement may have been better served by pre-op chemoradiation. Accrual of pts into clinical trials was low. Documentation of clinical findings, treatment toxicity and outcome was poor and might arguably be improved with tumour site-specialisation and multi-disciplinary team working.

**P56**

**PHASE I TRIAL OF ZD1839 ('IRESSA') IN COMBINATION WITH 5-FLUOROURACIL (5-FU) AND LEUCOVORIN (LV) IN PATIENTS WITH METASTATIC COLORECTAL CANCER.**

JS de Bono,[1*] LA Hammond,[1] J Figueroa,[2] L Schwartzberg,[3] L Ochoa,[1] A Patnaik,[1] N Olivo,[1] G Schwartz,[1] L Smith,[1] J Ochs,[4] EK Rowinsky.[1] [1]Institute for Drug Development, 7979 Wurzbach Road, Suite 400, Zeller Building, Cancer Therapy and Research Center, University of Texas Health Science Center, and Brooke Army Medical Center, San Antonio, TX; [2]Joe Arrington Cancer Center, Lubbock, TX; [3]Response Oncology, Inc., Memphis, TN; [4]AstraZeneca, Wilmington, DE, USA.

**Background:** Epidermal growth factor receptor (EGFR) signaling has been implicated in the proliferation and survival of cancer cells. ZD1839 ('Iressa') is an orally active selective inhibitor of EGFR signaling. Single-agent clinical trials have demonstrated promising antitumor activity in several tumor types. Combination studies with cytotoxic drugs, including 5-FU, have indicated additive or synergistic activity. **Methods:** Patients with metastatic colorectal cancer, not selected for EGFR status and treatment-naive in the metastatic setting, were treated in this two-part study of escalated intermittent and continuous schedules of ZD1839 plus 5-FU/LV administered utilizing the Mayo regimen (370/20 mg/m$^2$ daily for 5 days). **Results:** In part 1, 20 patients were randomized to intermittent ZD1839 plus 5-FU/LV on one of two schedules: (A) ZD1839 on days 1-14, 5-FU/LV on days 8-12 (cycle 1) and days 36-40 (cycle 2); or (B) 5-FU/LV on days l-5 (cycle l) and days 29-33 (cycle 2) plus ZD1839 on days 22-35. Intermittent ZD1839 dose-escalation (250/400/500 mg) was carried out in cohorts of 6-12 patients. In part 2, continuous ZD1839 was administered at 500 mg, the highest safely administered dose in part 1, with 5-FU/LV on days 8-12 (cycle l) and days 36-40 (cycle 2). One patient had dose-limiting G3 ataxia at 500 mg on the intermittent schedule, and one patient had dose-limiting G3 skin rash at 500 mg on the continuous schedule. G3/4 toxicities included neutropenia (9

 © 2002 Cancer Research UK

patients). Gl/2 toxicities included rash, diarrhea, mucositis and neutropenia. Pharmacokinetic studies indicate no significant change in mean exposure to either ZD1839 or 5-FU/LV when they are given in combination. One patient had a complete response; 5 partial responses were observed and 12 patients had durable stable disease. The overall median (range) duration on study was 70 (13-347) days. **Conclusion:** This combination of continuous ZD1839 with Mayo regimen 5-FU/LV is well tolerated and demonstrates promising activity. Further trials with ZD1839 in colorectal cancer are now planned or underway.
'Iressa' is a trademark of the AstraZeneca group of companies.

**P57**

**ENDOSCOPIC PALLIATIVE TREATMENT: ANALYSIS OF 948 CONSECUTIVE PATIENTS IN THE SCOTTISH AUDIT OF GASTRIC AND OESOPHAGEAL CANCER.**

AM Thompson*, R Stuart for the Scottish Audit of Gastric and Oesophageal Cancer, University of Dundee, DD1 9SY.

**Aim**: To document current practice, seek variations in practice and record complications from Endoscopic Palliative Treatment (EPT) in a population cohort of patients with oesophageal or gastric cancer.
**Methods**: Data was collected from every patient diagnosed with oesophageal, junctional or gastric cancer in Scotland over a 2 year period (to September 1999) with subsequent minimum 1 year follow up. Ethical consent was obtained for the study.
**Results**: EPT was given to 948/3,293 patients, predominantly with oesophageal or junctional cancers. Stent alone (506 patients) or LASER alone (117 patients), combination approaches including radiotherapy (188 patients) or chemotherapy (134 patients) were used. The majority of patients (>65%) had normal physical activity / only strenuous activity restricted before undergoing EPT. The consultant looking after the patient considered stents were used appropriately for 95% of patients (for grade 3 or 4 dysphagia) and LASER in 83% of patients (for grades 1, 2, 3 dysphagia). There was significant variation in delivery of EPT by healthboard of residence (chi square test, P<0.001), but not by deprivation quintile.
Complications, recorded in 221/948 patients (23%), were associated with multiple treatments (P<0.001). Oesophageal perforation was documented in 23patients (4%) at the time of stent placement and 3 patients (2%) after LASER.
Survival for all patients receiving EPT was 40% at 6 months, 17% at 12 months, 10% at 18 months and 6 % at 24 months, suggesting benefits from EPT even in patients with advanced disease.
**Conclusion**: Despite potential differences between patients receiving different types of EPT, there is evidence for regional variation in the approaches used. However, evidence from this population based cohort demonstrates that appropriate use of intervention may provide prolonged palliation for patients with upper gastrointestinal cancer.

**P58**

**THE ROLE OF VITAMIN C IN THE PATHOGENESIS OF BARRETT'S OESOPHAGUS**

[1]Fountoulakis, A., [1]Martin, I.G., White, K.L.M., [2]Cade, J., [1]Sue-Ling, H.M. and *Wild C.P.
Molecular Epidemiology Unit, Division of Epidemiology and Health Services Research, [1]Academic Unit of Surgery and [2]Nuffield Institute for Health,
School of Medicine, University of Leeds, Leeds LS2 9JT, UK.

Barrett's oesophagus (BO) is a condition where the squamous epithelium of the lower oesophagus is replaced by a metaplastic, specialised columnar type. BO arises as a complication of gastro-oesophageal reflux disease and is associated with a 30-125 fold increased risk of oesophageal adenocarcinoma (OA) development. Over the last 30 years the incidence of OA has increased dramatically, implicating environmental factors in the aetiology of this disease. Recent epidemiological studies have linked low fruit and vegetable consumption and dietary antioxidants with increased OA risk. The aim of this study was to test the hypothesis that levels of antioxidants are reduced in the plasma and modified mucosa of BO patients.
Following ethical approval, blood samples and endoscopic biopsies (squamous, Barrett's and gastric mucosa) were obtained from 57 BO and an equal number of age and sex matched control (blood sampled only) patients. BO patients also completed food frequency questionnaires. Plasma concentrations of vitamins A, C and E were measured by HPLC and compared between BO and control patients. Within BO patients, levels of vitamin C were also compared across different mucosal sites.
Histology revealed intestinal metaplasia in 45, gastric metaplasia in 9 and dysplasia in 3 of the BO patient group. Total vitamin C (and ascorbic acid levels) were significantly (p<0.05) reduced in the plasma of patients with intestinal metaplasia (8.3 [5.6-11.2] μg vit. C/ml {median with interquartile range}), compared with matched control patients (10.4 [6.4-14.5] μg vit. C /ml). There was no significant difference in the plasma levels of vitamins A and E between these two groups.
A positive association was identified between total vitamin C levels and dietary intake of vitamin C in BO patients. Tissue levels of total vitamin C (and ascorbic acid) were significantly (p<0.05) lower in Barrett's (96.0 [75.6-116.6] μg vit. C/g) compared with matched squamous mucosa (118.3 [88.5-156.3] vit. C μg/g), whereas levels measured in Barrett's and gastric (81.6 [68.0-102.7]μg/g) mucosa were similar.
In conclusion, levels of vitamin C are reduced in the plasma and specialised columnar epithelia of BO patients. This antioxidant may play an important role in the pathogenesis and neoplastic progression of BO, perhaps by modulating the extent of oxidative stress and associated DNA damage in oesophageal mucosa.

**P59**

**THE ROLE OF DNA DAMAGE IN THE DEVELOPMENT OF BARRETT'S OESOPHAGUS AND OESOPHAGEAL ADENOCARCINOMA**

Olliver J.R*., Hardie L.J., [1]Clark, G.W.B., Dexter S.,[2]Chalmers, D. and Wild C.P.
Molecular Epidemiology Unit and [1]Department of Surgery, School of Medicine, University of Leeds and [2]Gastroenterology Unit, Leeds General Infirmary, Leeds LS2 9JT, UK

Oesophageal adenocarcinoma (ADC) is increasing in incidence in the Western world. Survival is poor with patients having a median survival time of less than a year. The strongest risk factor (30-125 fold increase) for the development of ADC is the presence of the precursor condition, Barrett's oesophagus (BO), where the squamous epithelium is replaced by a metaplastic specialised columnar epithelium, usually in response to gastro-oesophageal reflux (GOR).
Molecular changes which occur during the development of ADC may prove useful as markers to assess the progression from BO to ADC *(Bani-Hani et al, 2000; JNCI 92;1316-1321*). It is our hypothesis that these molecular changes may result from DNA damage caused by GOR. This study therefore aims to examine whether GOR is associated with an elevation in DNA damage in BO. Ethical approval was granted for BO and control patients to be recruited from local dyspepsia and Barrett's surveillance endoscopy clinics. For each BO patient, biopsies were collected from normal squamous oesophageal, gastric and Barrett's mucosa. Control patients had only normal squamous tissue sampled. In addition, questionnaires were completed detailing reflux history, medication, smoking and alcohol intake. Biopsies were subject to DNA damage analysis using the alkaline 'Comet' assay to measure levels of DNA strand breaks (SB).
Preliminary findings indicate a significantly raised (P<0.001) mean level of SBs in BO tissue (28.32±5.70 {mean % tail DNA±SD}, N=18) when compared with matched squamous mucosa (19.29±6.61) from the same patient. Out of 18 Barrett's patients, 16 showed higher levels of DNA damage in BO tissue compared to squamous mucosa. In contrast there was no significant difference (P>0.05) in the mean levels of SBs when comparing BO (28.32±5.70) and gastric mucosa (26.71±9.01), perhaps reflecting the histological similarity between these tissues. Somewhat surprisingly, levels of SBs in the squamous tissue of control patients (25.73±5.87) were higher than those in the squamous tissue of BO patients (19.29±6.61). There was no association between DNA damage levels and age, sex, *Helicobactor pylori* infection, alcohol or smoking.

In conclusion, our observations to date support the hypothesis that higher levels of DNA damage occur in BO tissue compared to squamous mucosa. This is consistent with a role for oxidative DNA damage in the development of ADC.

*This work is supported by Yorkshire Cancer Research*.

**P60**

### ABSTRACT WITHDRAWN

**P61**

**ADJUVANT TREATMENT OF DUKES'S C COLORECTAL CANCER WITH 6 CYCLES (10 WEEKS) OF MODIFIED DeGRAMONT 5-FLUOROURACIL/ FOLINIC ACID PRODUCES DISEASE FREE AND OVERALL SURVIVAL COMPARABLE WITH 6 MONTHS OF TREATMENT**

Russell D. Petty*, Les Samuel and Jim Cassidy.
Department of Oncology, ANCHOR UNIT, Aberdeen Royal Infirmary, Aberdeen.

Randomised controlled trials have established the benefit of adjuvant treatment with 5-Fluorouracil/Folinic acid (5-FU/FA) for patients with Duke's C Colorectal cancer (CRC)[1]. However, the optimal scheduling of treatment remains to be determined. Clinical trials have determined that 6 months of treatment with a bolus or infusional regimen produces results similar to longer periods[1].Breast cancer trials have collected the most data on adjuvant chemotherapy. Overall, for the adjuvant treatment of node positive breast cancer, meta-analyses suggest that the benefit is similar to that seen in Dukes C CRC with 5-FU/FA- an approximately 25-30% reduction in the relative risk of recurrent disease and death[2]. Randomised controlled trials in breast cancer have shown that treatment with 3 or 4 cycles of treatment ( 6-9 weeks) provides similar disease free survival (DFS) and overall survival (OS) compared with longer periods of treatment[3]. Such results may be applicable to the adjuvant treatment of Duke's C CRC.

Here we present the results of a consecutive cohort of 42 patients with curative resection of Dukes C CRC treated adjuvantly with 6 cycles (10 weeks) of modified De Gramont chemotherapy (FA 200mg/m$^2$ 2 hour infusion day 1 and 2, 5-FU 600mg/m$^2$ bolus day 1 and 2, , 5-FU 600mg/m$^2$ 22 hour infusion day 1 and 2, repeated every 2 weeks). All patients commenced treatment between January 1998 and February 1999 within 6 weeks of surgery- 25(60%) were male 17(40%) female, median age 62 years (range 39-78). All patients were WHO performance status 0-1, there were 27(65%) rectal cancers, 8(19%) sigmoid cancers, 5(11%) left colon cancers, and 2(5%) right colon cancers. There were 37(88%) patients with Dukes C1, 5(12%) Dukes C2, and there were 1-4 lymph nodes involved in 27(64%), 5 or more lymph nodes involved in 13(31%). The treatment was well tolerated with grade 3 toxicity (mostly diarrhoea) in 11 (26%) and grade 4 in only 3(7%).

Median follow up for patients still alive was 37 months. Estimated 3 year DFS was 71.6% and OS was 77.7% (life table analysis). Kaplan-Meier analysis reveals that median DFS and OS have not yet been reached. These results are comparable with those found in randomised trials of 6 months of 5-FU/FA with both infusional and bolus type regimens[1]. Confirmation of these results in larger studies (we are currently collecting data on a larger cohort) may allow the routine use of shorter (10 weeks) periods of adjuvant treatment with 5-FU/FA for Duke's C CRC- this is likely to be beneficial for quality of life outcomes, toxicities of treatment and in terms of pharmacoeconomics. Such data may also prove applicable to adjuvant treatment with new 5-FU oral pro-drugs, and in 5-FU combination chemotherapy.

1.Polychronis A and Haller D 2000, The Effective Management of CRC p3.
2.Early Breast Cancer Trialist Collaborative Group 1992 Lancet 339 (8785): 71.
3. Hortobgyi GN, Seminars in Oncology2001 28(5) Suppl 16:33.

**P62**

**LOCALLY ADVANCED PANCREATIC CANCER TREATED WITH RADIATION AND 5-FLUOROURACIL,**

S. Mawdsley*[1], M. Hall[2], R. Glynne-Jones[2]. [1]The Gray Cancer Institute & [2]Marie Curie Research Wing, Mount Vernon Hospital, Northwood, Middlesex HA6 2RN, UK.

**Introduction**: Current approaches to the treatment of pancreatic cancer are disappointing, with few patients surviving longer than 12 months from the time of diagnosis. This report describes the survival and local relapse rates of patients with locally advanced pancreatic carcinoma, treated at Mount Vernon Hospital with 5-fluorouracil (5FU) chemoradiation. Locally advanced was defined by the extent of lymphadenopathy or invasion of adjacent organs, without evidence of distant metastases.

**Methods**: Between 1994 and 2000, 24 patients were treated with radiation and 5-fluorouracil (5-FU). All patients were WHO performance status 0-2 and had pre-treatment radiological staging with CT or MRI. The radiotherapy was CT planned and the standard dose prescribed was 45Gy in 25 fractions over 5 weeks. 5-FU was given as a 60 minutes infusion on days 1-5 and 29-33 of the radiotherapy, at 350mg/m$^2$, following low dose folinic acid. Actuarial survival, local control and toxicity rates according to WHO criteria, were assessed for the group.

**Results**: The median survival was 12 months, with a 48% 1-year survival and a 29% 2 year survival. The median time to progression was 8 months. The treatment was well tolerated and all patients achieved 100% compliance. There were no grade 3-4 haematological toxicities. 30% experienced diarrhoea but in all cases this was mild (grade 1-2). 58% experienced emesis and in 3 patients this was grade 3, however no interruption of treatment was required. In terms of radiological tumour response, 5 patients (22%) had a complete response, 10 patients (45%) demonstrated partial tumour shrinkage and a further 3 additional patients had radiological stable disease. The majority of patients (92%) experienced a symptomatic improvement.

**Conclusion**: Chemoradiation with 5-fluorouracil can produce effective local control with symptomatic improvement in patients with localised carcinoma of the pancreas. This is not at the expense of considerable toxicity, either late or acute.

© 2002 Cancer Research UK

**P63**

**THE GASTRIC CANCER 5 (GC5) STUDY: ANTIBODY RESPONSE AND SIDE EFFECT PROFILE OF G17DT 500mcg IN POST GASTRIC CANCER RESECTION PATIENTS**

EJ Dickson*, E Cowan, RC Stuart.
University Dept of Surgery, Glasgow Royal Infirmary, Glasgow.

**AIM:** Gastrin has a trophic effect on gastrointestinal epithelium as part of its normal physiological function, and gastrin 17 in particular stimulates the growth of gastrointestinal (GI) cancer cell lines. The aims of the GC5 study were to determine the anti-gastrin antibody response to G17DT 500mcg (Aphton Corps) and to evaluate patient tolerance at this higher dose.
**METHODS:** Gastrin 17 linked to the diphtheria toxoid (G17DT) was administered as a 500mcg intramuscular injection at weeks 0, 2 and 6 to seven patients who had previously undergone potentially curative gastric cancer resection. Approval was obtained from the local Ethics Committee. G17DT antibody titres were measured over the follow up period (median 419 days, range 391-463 days). Patients underwent CT scanning for assessment of tumour recurrence on 3 occasions.
**RESULTS:** All seven patients achieved an antibody response (median 177.2 units, range 33.9-313.7 units) which remained quantifiable in five patients at the end of the follow-up period, although the third dose was administered to one patient only. Four patients developed an abscess at the injection site and all seven patients reported minor to severe local side effects (pain, immobility, and tenderness). Based on CT scanning no patients had tumour recurrence during this period. The median length of time from surgery to most recent follow-up is 1148 days (range 790 to 1716 days). All patients are currently disease-free.
**CONCLUSIONS:** At this dosage schedule G17DT is an effective method of producing a sustained anti-gastrin antibody response at the expense of unacceptable tolerance. However the high dose of 500mcg may be utilised in conjunction with chemotherapy as combination therapy, as the chemotherapeutic agents are anti-inflammatory and may suppress injection site reactions. Of note, there is no evidence of local nor systemic recurrence in any patient.

**P64**

**IMPAIRED POST-OPERATIVE NEUTROPHIL LEUCOCYTOSIS AND ACUTE COMPLICATIONS FOLLOWING SHORT COURSE PREOPERATIVE RADIOTHERAPY FOR OPERABLE RECTAL CANCER**

Hartley A*[1], Giridharan S[1], Srihari N[1], McConkey C[2] and Geh JI[1]
[1]Cancer Centre, Queen Elizabeth Hospital, Birmingham.B15 2TH
[2]Clinical Trials Unit, CRC Institute of Cancer Studies, Birmingham

**Introduction:** In the Dutch Colorectal Cancer Group Trial the 180-day mortality was increased in patients undergoing total mesorectal excision (TME) more than 3 days after the completion of short course preoperative radiotherapy (SCPRT). A similar relationship between time from the first day of SCPRT to surgery (overall treatment time (OTT)) and acute complications was observed in a recent retrospective audit. One proposed explanation for these findings and the relationship of acute complications with age and radiation volume is suppression of the neutrophil leucocytosis normally seen following abdominal surgery. This study aimed to determine the relationship between acute complications within 3 months of SCPRT and perioperative absolute neutrophil counts (ANC).
**Method:** A SCPRT database of 176 patients treated in 1998 and 1999 was used. Preoperative (preop) ANC (48 hours before surgery) and postoperative (postop) ANC (48 hours after surgery) were obtained from haematology archives. A two sample Wilcoxan test was used to compare preop ANC, postop ANC and the ratio postop ANC/preop ANC (ANC ratio) in patients with and without complications. The relationship between these parameters and OTT, age and radiotherapy field length was examined.
**Results:** Due to pre-clerking preop ANC was only available in 83 (47%) patients. Postop ANC was performed in 128 (73%) patients. ANC ratio was calculable in 72 (41%) patients. There was no significant association between acute complications and preop ANC (p=0.25) or postop ANC (p=0.45). ANC ratio was significantly higher in patients without complications (p=0.02). There was no clear relationship between ANC ratio and OTT, field length or age. Logistic regression including ANC ratio, OTT, age and field length showed that ANC ratio (p=0.02) and OTT (p=0.06) might be significant factors.
**Conclusions:** In this retrospective study there appears to be a relationship between the magnitude of postop neutrophil leucocytosis and the absence of acute complications following SCPRT. However, this study is limited by a majority of preoperative full blood counts being taken many days before surgery at pre-clerking.

**P65**

**PATIENTS' ATTITUDES TO CEA TESTING IN COLORECTAL CANCER FOLLOW-UP: A PILOT STUDY.**

NL Fernie*, MJ Mackean, Edinburgh Cancer Centre, Western General Hospital, Crewe Road South, Edinburgh EH4 2XU.

The routine use of CEA testing in the follow-up of patients who have undergone curative resection +/- adjuvant treatment for colorectal cancer remains a controversial issue. Unlike many other cancers, where metastatic disease is invariably incurable, there is a small but significant chance of detecting localised liver or lung metastases, resection of which can result in cure[1]. Approximately 25% of patients with up to 3 resectable liver metastases are long term survivors. Randomised trials have, however, failed to show an overall survival benefit from CEA testing. The cost-effectiveness of this practice has also been called into question, given the common nature of the disease and the expense of diagnosing a recurrence that is often incurable[2].
The attitudes of colorectal cancer patients to follow up and its effect on their quality of life have been previously studied. In general patients reported a strong preference for follow-up, even if it did not lead to earlier detection of recurrence and despite any anxieties provoked by the follow-up visit[3]. The attitudes of patients towards specific investigations, notably the CEA blood test, have not been formally examined.
In this ongoing pilot study, patients' attitudes to and preferences for CEA testing are being assessed using a questionnaire. 12 patients have been recruited so far, 7 male and 5 female. Median age 61-70 years. 8 of the patients questioned were within 2 years of their surgery and all were within 5 years of resection. 9 of the 12 patients admitted to worrying to some degree about cancer recurrence. 2 of the patients questioned had had CEA detected recurrences. 6 patients worried a little or somewhat about their CEA result after the clinic whilst 6 were not at all concerned. 11 patients would accept the test even if there were a significant risk of a false positive or false negative result. All patients recruited wished to know if an asymptomatic recurrence were detected in this way, even if incurable.
Early results of this pilot study of patient preferences for CEA testing indicate that an overwhelming majority of patients are in favour of the test. This is despite uncertainties as to the accuracy of the result and the generally low cure rate of recurrences, even when detected early in this setting. It is our view that in deciding whether to test for CEA we should be guided our patients' preferences in addition to published data on survival and health economics.

1. Registry of Hepatic Metastases (1988) Resection of the liver for colorectal carcinoma metastases. *Surgery* 103: 278
2. Nelson RL (1995) Postoperative evaluation of patients with colorectal cancer. *Sem Oncol* 22 (5): 488
3. Stiggelbout AM, de Haes JCJM, Vree R *et al* (1997) Follow-up of colorectal cancer patients: Quality of life and attitudes towards follow-up. *Br J Cancer* 75 (6): 914

**P66**

**SELECTIVE PRE-OPERATIVE RADIOTHERAPY FOR RECTAL CANCER, A RETROSPECTIVE AUDIT OF RESULTS**

Mark A Potter*, Catriona McLean, Hamish A Phillips
Departments of Surgery* and Oncology Wetern General Hospital, Edinburgh

Preoperative radiotherapy for rectal cancer reduces local recurrence but has a limited impact on overall survival. A selective policy of preoperative radiotherapy for patients at high risk of local recurrence is pursued at our institution. Indications for short course preoperative radiotherapy are low cancers, perirectal infiltration on imaging, tethering and anterior cancers close to the prostate. The results for 1996 – 2000 have been audited.

© 2002 Cancer Research UK

